# Sodium-glucose co-transporter 2 inhibitors in left ventricular assist device and heart transplant recipients: a mini-review

**DOI:** 10.1007/s10741-024-10465-z

**Published:** 2024-11-08

**Authors:** Emyal Alyaydin, Danaë Parianos, Julia Hermes-Laufer, Matthias P. Nägele, Liesa Castro, Maria Papathanasiou, Holger Reinecke, Andreas J. Flammer

**Affiliations:** 1 Department of Cardiology, University Hospital Zurich, Zurich, Switzerland; 2 Department of Cardiovascular Surgery, University Heart and Vascular Center Hamburg, Hamburg, Germany; 3 Department of Cardiology, Angiology and Intensive Care Medicine, Goethe University Hospital, Frankfurt, Germany; 4 Department of Cardiology I - Coronary and Peripheral Vascular Disease, Heart Failure, University Hospital Muenster, Muenster, Germany

**Keywords:** Left ventricular assist device, Heart failure, Heart transplantation, Sodium-glucose co-transporter 2 inhibitor, Diabetes mellitus

## Abstract

In recent years, sodium-glucose co-transporter 2 inhibitors (SGLT2i) emerged as promising therapeutic agents in managing heart failure (HF). They demonstrated a significant impact on reducing HF hospitalizations and related mortality in patients with reduced and preserved ejection fraction. However, evidence supporting their use in patients with left ventricular assist device (LVAD) and heart transplant (HT) recipients is still limited. We identified six key studies investigating the safety and efficacy of SGLT2i in LVAD and HT recipients. In patients with LVAD, prescription of SGLT2i was predominantly associated with improved fluid management and reduced pulmonary artery pressures. However, the results regarding their effects on body weight, hemoglobin A1c, diuretic use, and right ventricular function were contradictory. In terms of safety, SGLT2i were generally well-tolerated in the LVAD population, and the reported incidence of adverse events was low. In HT recipients, SGLT2i were associated with better glycemic control and weight reduction. No relevant adverse events were reported. Despite these encouraging results, the long-term safety and efficacy of SGLT2i in these vulnerable patient populations are yet to be investigated. Future randomized controlled trials are needed to address existing gaps in evidence and help integrate SGLT2i into clinical practice for LVAD and HT recipients.

**Graphical abstract:**

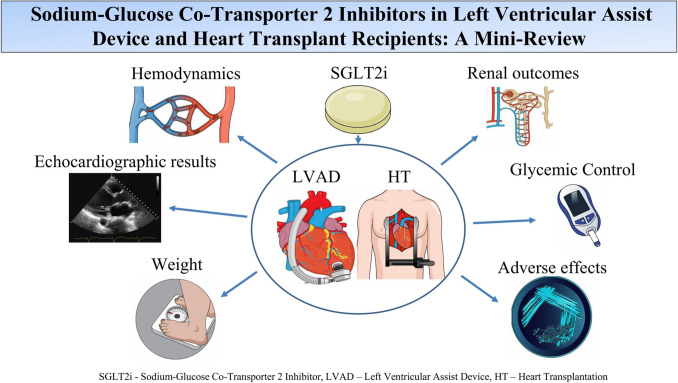

## Introduction

Sodium-glucose co-transporter 2 inhibitors (SGLT2i) have emerged as promising agents in managing heart failure (HF). Landmark trials such as DAPA-HF and EMPEROR-Reduced demonstrated their role in reducing HF hospitalizations and cardiovascular mortality among patients with reduced left ventricular ejection fraction (LVEF), irrespective of diabetes status [1, 2]. The EMPEROR-Preserved and DELIVER trials have been pivotal in further expanding the indications for SGLT2-i across the whole spectrum of LVEF [3, 4].

The promising cardioprotective properties of SGLT2i appear to extend beyond glycemic effects and include blood pressure reduction, improvement in myocardial energetics, and modulation of inflammatory pathways [5–9]. This broad therapeutic profile suggests potential benefits in patients who were not included in the mentioned HF trials, such as in HF patients managed with left ventricular assist devices (LVADs) and heart transplantation (HT).

Patients with LVADs represent a unique population of HF patients. Despite technological advances and improved perioperative care, they remain at high risk for adverse cardiovascular events, such as right heart failure and arrhythmias [10, 11]. Similarly, HT recipients face a lifelong risk of allograft dysfunction and metabolic complications that can compromise graft and patient survival [12–14]. Emerging data suggest that SGLT2i may offer clinical advantages in these populations by mitigating residual risks and improving cardiovascular outcomes [15–20]. However, LVAD and HT patients a susceptible for infections due to immunosuppression and device-related factors, which may represent an ongoing challenge under treatment with SGLT2i [21, 22].

The current review aims to provide an analysis of the safety and efficacy of SGLT2i in LVAD and HT patients. Examining the latest research findings, we aim to elucidate the role of SGLT2i in these vulnerable patient populations.

## Methods

We conducted a literature review of primary studies examining adult HT recipients and patients with LVAD treated with SGLT2i as a monotherapy or in combination with other antihyperglycemic agents. We searched for articles published from May 5, 2020, the date of FDA approval of dapagliflozin for heart failure, until May 25, 2024. The databases included PubMed, Google Scholar, Ovid Embase, Ovid MEDLINE, and The Cochrane Library (Wiley). We used the following keywords in various combinations: “left ventricular assist device”, “ventricular assist device”, “heart transplantation”, “sodium-glucose co-transporter 2 inhibitor”, “sodium glucose co-transporter 2 inhibitor”, “SGLT-2 inhibitor”, and “SGLT2 inhibitor”. Due to the limited available evidence, all published observational studies were included. Case reports and conference abstracts were excluded. No interventional studies on this topic were identified. The primary clinical outcomes included the safety profile and efficacy of SGLT2i. The main focus was on their impact on body mass index (BMI), hemoglobin A1c (HbA1c), and furosemide dosage. Additionally, adverse effects and tolerability were assessed. In LVAD recipients, the studies also investigated the resulting changes in hemodynamic parameters and device settings.

### SGLT2 and patients with LVAD

#### Baseline characteristics

We identified four key observational studies investigating the impact of SGLT2i in LVAD patients [15–18]. Three were single-center studies (*n* = 560 patients), and one was conducted in a multicenter setting (*n* = 138). All four articles were published between 2022 and 2024. Overall, the proportion of patients on SGLT2i was low (*n* = 99, 14.2%). Among these, Empagliflozin was the most commonly utilized SGLT2i (*n* = 59, 59.6%), followed by Dapagliflozin (*n* = 22, 22.2%) and Canagliflozin (*n* = 2, 2.0%). One study did not specify which SGLT2i was used. The type of LVAD system was specified in three of the four studies, with HeartMate3 (Abbott Inc., Chicago, IL, USA) being the most commonly implanted device (*n* = 67, 67.7%). Most patients were treated with a bridge-to-transplant approach. The median reported follow-up ranged from 30 to 354 days. Most of the patients on SGLT2i were male (*n* = 82, 82.8%). Ischemic cardiomyopathy was the most common etiology of advanced HF (*n* = 54, 54.5%). The median body mass index (BMI) was between 26.8 and 33.6 kg/m^2^ in all three studies where it was reported. Approximately 80% of the patients on SGLT2i had diabetes mellitus (*n* = 79). The median baseline left ventricular ejection fraction (LVEF) ranged from 18.0 to 25.0%. According to the KDIGO classification of renal function, patients had renal insufficiency classified as G2–G3a in all three studies reporting the estimated glomerular filtration rate (eGFR) (Table 1).

**Table 1:** Baseline characteristics of LVAD patients on SGLT2i

**Study**	**Design**	**Patients on SGLT2i, n (%) / Study population (n)**	**SGLT2i, n (%)**	**LVAD type, n (%)**	**Therapeutic goal**	**Follow-up on SGLT2i**	** Age, years**	**Male, n (%)**	**Ischemic HF, n (%)**	**BMI**(kg/m^2^), mean ± SD	**Diabetes mellitus, n (%)**	**LVEF (%)**	**Creatinine, mg/dl**	**eGFR, ml/min/1.73 m**^2^	**INTERMACS, ≤ 4, n (%)**
Cagliostro M et al, 2022	Single centre observational study	34 (6.7) / 509	Empagliflozin, n = 22 (64.7) Dapagliflozin, n = 10 (29.4) Canagliflozin, n = 2 (5.9)	HVAD, n = 9 (26.5) HM2, n = 7 (20.6) HM3, n = 18 (52.9)	BTT, n = 11 (35.5) DT, n = 20 (64.5)	> 30 days	56.1 ± 10.6	27 (79.4)	17 (50.0)	33.6 ± 7.2	34 (100)	18.6 ± 10.4	1.4 ± 0.4	59.0 ± 19.0	34 (100)
Moady G et al, 2023	Single centre observational study	20 (100) / 20	Empagliflozin, n = 14 (70.0) Dapagliflozin, n = 6 (30.0)	HM3, n = 20 (100)	NR	6 months	64.7 ± 12.1	15 (75.0)	15 (75.0)	27.9 ± 4.6	20 (100)	25.0 ± 6.0	NR	64.8 ± 8.0	20 (100)
Chavali et al, 2023	Single centre observational study	16 (51.6) / 31	NR	NR	BTT, n = 31 (100)	101.5 days, IQR (35.7 – 190.8)	52.3 (46.7 – 57.9)	15 (93.8)	NR	NR	2 (12.5)	12.5 (10. – 20.0)	NR	71.9 ± 18.7	16 (100)
Fardmann et al, 2024	Multicentre observational study	29 (21.0) / 138	Empagliflozin, n = 23 (79) Dapagliflozin, n = 6 (21)	HM3, n = 29 (100)	BTT, n = 101 (73.2) DT, n = 37 (26.8)	354 days, IQR (206 - 786)	62.0 ± 6.7	25 (86.0%)	22 (76.0)	26.8 ± 3.8	23 (79.0)	18.0 ± 6.0	1.3 ± 0.5	NR	29 (100)

*SGLT2i *sodium-glucose co-transporter 2 inhibitors, *LVAD *left ventricular assist device, *HVAD *Heartware Ventricular Assist Device, *HM2 *HeartMate 2, *HM3 *HeartMate 3, *BTT *bridge-to-transplant therapy, *DT *destination therapy, *IQR *interquartile range, *NR *not reported

#### Outcomes

The studies on the use of SGLT2i in LVAD patients were heterogeneous and assessed different outcomes (Table 2).

**Table 2:** Clinical and Safety Outcomes in LVAD patients on SGLT2i

Study	Outcomes on SGLT2i
Cagliostro M et al, 2022	30-, 60-, and 180-days follow-up No significant change in BMI, HbA1c, or diuretic dose Difference noted in BUN at 180 days (27 ± 10mg/dl at baseline vs 25 ± 10mg/dl at 180 days, p = 0.049) Potential SGLT2i-related adverse events - 3 genitourinary infections, 2 episodes of acute kidney injury, and 2 limb amputations, 4 driveline infection No episodes of diabetic ketoacidosis, volume depletion, fracture, or hypersensitivity reactions
Moady G et al, 2023	Few suction events, without the need for pump speed adjustment No change in mean arterial pressure Modest decline in renal function in six patients within the first weeks after SGLT2i initiation No events of diabetic ketoacidosis or limb amputation
Chavali et al, 2023	No significant differences in renal function, weight, mean arterial pressure and right heart catheter derived intracardiac hemodynamics There were numerically lower infection-related (n = 4 vs 7, HR 0.32 (0.08–1.28), p = 0.11) and haemocompatibility-related (n = 3 vs 4, HR 0.52 (0.09–2.83), p = 0.45) adverse events in the SGLT2i group, albeit non-significant
Fardmann et al, 2024	Decrease in the daily dose of furosemide 47 to 23.5 mg/day (mean difference = 23.5 mg/d, 95% CI 8.2–38.7, p = 0.004) Significant weight reduction (mean difference = 2.5 kg, 95% CI 0.7–4.3, p = 0.008) Significant 5.6 mm Hg reduction in systolic pulmonary artery pressure (sPAP) was measured during RHC (95% CI 0.23–11, p = 0.042) in a subgroup of 11 (38%) patients. No adverse events were recorded during median follow-up of 354 days, IQR (206–786).

*LVAD *left ventricular assist device, *SGLT2i *Sodium-glucose co-transporter 2 inhibitors, *BMI *body mass index, *BUN* blood urea nitrogen, *HbA1c* hemoglobin A1c, *eGFR *estimated glomerular filtration rate

##### Fluid Management and Hemodynamics

Fardman et al. reported improvements in fluid management and pulmonary pressures in patients treated with SGLT2i, with a significant reduction in invasively derived systolic pulmonary artery pressure (PAP). Given that the maximum effect of left ventricular (LV) unloading on PAP reduction is usually achieved between 1 and 3 months post-surgery and the fact that SGLT2i were initiated at a median of 108 days (IQR 26–477) after LVAD implantation in this study, the authors hypothesized that the maximum effect of mechanical LV unloading on PAP reduction had already been reached [18, 23, 24]. Hence, they postulated that the observed further reduction in PAP could be attributed to the effects of SGLT2i. The beneficial effects of SGLT2i on PAP have already been investigated by previous studies, including the EMBRACE-HF trial [25, 26]. The proposed mechanisms for these effects include natriuretic synergism with loop diuretics. Accordingly, the authors reported a significant reduction in the diuretic dose in patients treated with SGLT2i. Moady et al. also observed a transient reduction in diuretic dose in patients treated with SGLT2i. They noted an increased number of suction events potentially attributable to enhanced diuresis during the first week of treatment [16]. SGLT2i block sodium and glucose reabsorption in the proximal tubule, but the duration and nature of their natriuretic and diuretic effects depend on the upregulation of SGLT2 and sodium-hydrogen exchanger 3 (NHE3), along with compensatory mechanisms in downstream nephrons. In euvolemic patients, counterregulatory sodium and water retention mechanisms activate more rapidly than in fluid-overloaded patients, limiting the duration of diuresis [27]. LVAD patients often have impaired renal function, and the glycosuria-related effects of SGLT2 inhibitors are reduced in patients with a glomerular filtration rate (GFR) below 45 mL/min. However, patients with chronic kidney disease still benefit from SGLT2 inhibitors [28]. While this evidence is encouraging, Cagliostro et al. and Chavali et al. did not find significant changes in diuretic dose with SGLT2i [15, 17]. Additionally, Chavali et al. did not observe any differences in the hemodynamic parameters after the initiation of SGLT2i [17].

##### Echocardiographic parameters

Despite the overall improvement in systolic PAP, Fardman et al. found an increase in the rate of right ventricular (RV) dysfunction. Severe RV dysfunction was observed in *n* = 3 (14%) of patients at follow-up, whereas none had severe RV dysfunction at baseline.This rate is comparable to the 8–11% reported in the literature [29, 30]. Additionally, *n* = 2 (9.5%) of the patients had dilated RV at baseline, compared to *n* = 7 (33.3%) at follow-up. The authors report that the assessment of the RV function was performed visually by experienced sonographer but provide no further details regarding the modality of the analysis. Despite the observed deterioration of the RV function, there was no difference in the invasively derived right atrial pressure (RAP) 6 months after the initiation of SGLT2i. Additionally, none of the patients with severe RV dysfunction required hospitalization for HF during the study period [18]. The discrepancy between echocardiographic findings and clinical outcomes warrants further investigation.

##### Renal outcomes

Moady et al. observed a transient worsening of renal function after initiation of SGLT2i, as evidenced by a drop in eGFR of ≥ 10% from baseline. This decline was temporary and stabilized after 2–3 months of therapy [16]. The initial reduction in eGFR is attributed to the modest reduction in blood pressure seen with SGLT2i, but it does not indicate poor long-term renal outcomes [31–33]. Additionally, all other studies reported no significant changes in renal function throughout their respective observational periods.

##### Glycemic Control and Weight

This is a critical outcome for LVAD patients with diabetes mellitus. Improved glucose management can help reduce the risk of complications like infections and enhance overall health [34]. Cagliostro M et al. did not observe significant changes in BMI or HbA1c. In contrast, Fardman A et al. reported a significant weight reduction, while Moady G et al. noted a significant decrease in HbA1c following the initiation of SGLT2i. Chavali S et al. found no substantial changes in body weight [15–18]. In a meta-analysis on diabetic patients, SGLT2i reduced HbA1c by 0.62% (95% CI -0.66 to -0.59) and body weight by 0.60 kg (95% CI -0.64 to -0.55). Moreover, the EMPA-REG trial demonstrated that the lowest dose of empagliflozin (10 mg) offers comparable benefits in lowering HbA1c, body weight, blood pressure, and reducing total and cardiovascular mortality as the highest dose (25 mg) [35]. While SGLT2i have shown notable improvements in HbA1c and weight in the general population, studies on their metabolic effects in LVAD patients have yielded inconsistent results.

##### Adverse events

Across all studies, the incidence of adverse events was generally low. Regarding safety and tolerability, the study by Cagliostro M et al. identified several adverse events potentially attributable to SGLT2i, including genitourinary infections, acute kidney injury, limb amputations, and driveline infections (DLI). Similarly, Fardman A et al. observed increased RV dysfunction in some patients but found no significant overall adverse effects linked to SGLT2i. In contrast, Chavali S et al. reported that the SGLT2i therapy was safe and well-tolerated. Moady G et al. also found that SGLT2i were well-tolerated with only minor adverse effects [15–18].

Regarding driveline infections and SGLT2i use, we acknowledge that current data from non-immunosuppressed populations do not show a significant increase in non-genital skin and soft tissue infections with SGLT2i therapy. A post-hoc analysis of the CANVAS and CREDENCE trials found no difference in non-genital infection rates between canagliflozin and placebo (HR 0.97, 95% CI 0.85–1.11; *P* = 0.70), and no significant increase in non-genital fungal infections either [36]. Thus, we do not have suffitient evidence regarding the risk of skin infections or potential driveline infections in LVAD patients. Additionally, due to the lack of evidence in immunosuppressed patients means we cannot definitively rule out the possibility of a higher risk in HT recipients. This is an important area for future research. Meanwhile, the glycemic improvement associated with SGLT2i is expected to reduce infection risk by improving overall metabolic control, which should be considered in assessing their benefit-risk profile [34].

##### Clinical implications and future directions

The evidence suggests that SGLT2i could play a vital role in managing LVAD patients, particularly those with concurrent diabetes. The current studies indicate potential benefits in glycemic control, fluid management, and hemodynamic stability. Across all studies, SGLT2i therapy was generally well-tolerated, with no significant increase in adverse events such as infections or ketoacidosis. This is particularly noteworthy given the complex and high-risk nature of LVAD patients, who often require multiple medications and have significant comorbidities.

### SGLT2 in HT recipients

 The safety and efficacy of SGLT2i in HT recipients have been explored in two pivotal studies [19, 20]. Both reports provide valuable insights into the efficacy and safety of SGLT2i in this specific patient population, although they use different methodologies (Table 3).

**Table 3:** Baseline Characteristics of Heart Transplant Patients on SGLT2i

Study	Design	Patients on SGLT2i, n (%) / Total patient population (n)	SGLT2i, n (%)	Immunosupression, n (%)	Follow-up on SGLT2i (months)	Time since HTx at baseline (years)	Age, years	Male, n (%)	Ischemic HF, n (%)	BMI (kg/m^2^), mean ± SD	Diabetes mellitus, n (%)	HbA1c (%)	Creatinine, mg/dl	eGFR, ml/min/1.73 m^2^
Cehic et al, 2019	Single centre observational study	22 (21.8) / 101	Empagliflozin, n = 22 (100)	Tacrolimus, n = 11 (50.0) Cyclosporine A, n = 6 (27.3) Everolimus, n = 10 (45.5) Prednisone, n = 10 (45.5)	> 12	5.0, IQR (2.0 – 12.3)	59.3 ± 11.9	17 (77.3)	NR	30.3 (25.6 – 32.4)	22 (100)	7.5 (6.5 – 8.2)	1.5 (1.1 – 1.7)	48 (43 – 61)
Sammour et al, 2021	Single centre observational study	5 (24) / 21 at baseline 17 (81.0) / 21 during the study	Empagliflozin, n = 5 and n = 21 (100), respectively	Tacrolimus, n = 21 (100) Sirolimus, n = 12 (57.0) Mycophenolate, n = 8 (38.0) Prednisone, n = 7 (33.0)	> 9	5.7, IQR (1.5 – 9.6)	59.4 ± 9.5	14 (66.7)	7 (33.3)	36 ± 5.1	21 (100)	7.5 (7.1 - 8.5)	1.3 (1.1 - 1.6)	55 (44 - 64)

*HT *heart transplantation, *SGLT2i *sodium-glucose co-transporter 2 inhibitors, *HTx *heart transplantation, *IQR *interquartile range

#### Baseline characteristics

Both studies were retrospective observational studies. All patients on SGLT2i (*n* = 39, 32%) were treated with empagliflozin. Most of these patients were male (*n* = 31, 86.1%), and the median follow-up period was 9.1 months in the study by Cehic et al. and 12 months in the study by Sammour et al. The median age was 59 years in both studies. The etiology of advanced HF prior to HT was not consistently reported. However, about a third of the patients in the study by Sammour et al. had a history of ischemic cardiomyopathy (*n* = 7, 33.3%). All patients had diabetes. In both studies, patients had renal insufficiency classified as G3a according to the KDIGO classification of renal function (Table 3).

#### Outcomes

Both studies included in this review examined similar outcomes, specifically focusing on the safety and efficacy of the SGLT2i (Table 4).

**Table 4:** Clinical and safety outcomes in heart transplant patients on SGLT2i

Study	Outcomes on SGLT2i
Cehic et al, 2019	Significant reduction in mean body (2 kg, p = 0.003, mean reduction 4.7 ± 5.7 kg) weight and mean BMI (1.3 kg/m2, p = 0.004, mean reduction 1.6 ± 2.0) Significant reduction in median furosemide dose Reduction of HbA1c (mean reduction 6.6 mmol/mol) No significant changes in renal function Three adverse events: exacerbation of urinary symptoms, dizziness, acute kidney injury No genitourinary infections
Sammour et al, 2021	Reduction in daily insulin requirements and the proportion of patients requiring insulin Significant reduction of HbA1c (7.5% at baseline vs 6.6% at follow up, p < 0.001) Significant reduction in mean body weight (247.3 ± 39.5 at baseline vs 220.1 ± 35.5 lbs at follow up, p < 0.001) and mean BMI (36 ± 5.1 at baseline vs 32 ± 4.3kg/m2 at follow up, p < 0.001) Significant reduction in low-density lipoprotein-cholesterol (65 (65.5 - 72) vs 57 (49.5 - 61.5) mg/dl, p = 0.006) No difference in eGFR No episodes of diabetic ketoacidosis, pancreatitis, or genital mycotic infections No drug interactions with immunsupressants

*SGLT2i *Sodium-glucose co-transporter 2 inhibitors, *BMI*  body mass index, *HbA1c *hemoglobin A1c, *eGFR *estimated glomerular filtration rate

##### Fluid Management and Hemodynamics

The treatment with empagliflozin led to a significant reduction in the median furosemide dose in the study by Cehic et al., with no significant change in systolic or diastolic blood pressure [19]. The study by Sammour et al. did not specifically report on fluid management or hemodynamic parameters [20]. However, the significant reductions in insulin requirements and body weight across the study population might indirectly influence fluid status. Insulin is known to stimulate sodium retention. Studies in diabetic models demonstrate that maintaining baseline insulin levels, even in the presence of hyperglycemia, can counteract natriuresis; thus, chronic hyperinsulinemia may lead to sustained sodium retention, particularly in the context of insulin resistance and metabolic disorders [37].

##### Echocardiographic parameters

The studies provided no data regarding echocardiographic parameters or their changes in the assessed HT populations.

##### Renal outcomes

In both studies, renal function remained stable in patients treated with empagliflozin compared to those on other glucose-lowering medication, highlighting the safety of empagliflozin with respect to renal function [19, 20]. However, a more extended observation period is needed to determine whether SGLT2i also offer renal protection for patients without diabetes mellitus. Recent evidence from the EMPA-KIDNEY trial demonstrates that empagliflozin promotes renal protection in patients with chronic kidney disease (CKD), irrespective of diabetes status. In a large population of patients with eGFR ranging from 20 to 45 ml/min/1.73 m² empagliflozin significantly reduced the risk of progression of kidney disease or death from cardiovascular causes compared to placebo, with consistent results across both diabetic and non-diabetic subgroups. Given these findings, further research is warranted to fully elucidate the long-term renal benefits of SGLT2i in HT patients without diabetes [28].

##### Glycemic Control and Weight

Overall, treatment with SGLT2i resulted in significant weight reduction and improved glycemic control. In the study by Cehic et al., empagliflozin treatment led to a decrease in body weight (median reduction of − 2.0 kg) and a mean reduction in HbA1c of 2.8% (6.6 mmol/mol) [19]. Similarly, Sammour et al. found that combining GLP-1 receptor agonists (GLP1-RAs) and SGLT2i significantly decreased body weight and HbA1c (from 7.5 to 6.6%) [20]. Additionally, there was a significant reduction in insulin requirements.

##### Adverse effects

Empagliflozin was well tolerated in the study by Cehic et al., with only three adverse effects reported: exacerbation of urinary symptoms, dizziness, and acute kidney injury. Notably, no genitourinary infections were observed in the empagliflozin group [19]. Similarly, the study by Sammour et al. reported no incidents of diabetic ketoacidosis, pancreatitis, or genital mycotic infections among patients using GLP-1RAs and SGLT2i [20]. Additionally, there were no noted drug interactions with immunosuppressive therapies.

##### Clinical implications and future directions

Both studies underscore the safety and efficacy of SGLT2i, alone or in combination with other antiglycemic agents for managing diabetes in HT recipients. Cehic et al. report that empagliflozin therapy is associated with potential benefits in improving body weight and glycemic control, while highlighting its safety in this population [19]. Similarly, Sammour et al. demonstrate that combining GLP-1 RAs and SGLT2i leads to significant improvements in insulin requirements, HbA1c, and body weight, suggesting the effectiveness of these therapies in managing HT recipients with diabetes [20]. However, there is a substantial gap in research concerning the role of SGLT2i in the vulnerable population of HT recipients. To address this, randomized controlled trials are needed to further validate the safety and efficacy of SGLT2i, both alone and in combination with other antiglycemic agents. Long-term studies are needed to evaluate their influence on renal function and metabolic complications following HT. Additionally, research should assess the impact of these therapies on transplant-related mortality, graft function, rejection episodes, coronary allograft vasculopathy, and the progression of chronic kidney disease. Currently, a phase 3 randomized placebo-controlled trial is ongoing, which evaluates the protective effects of dapagliflozin on renal function in patients after HT (DAPAHRT trial; ClinicalTrials.gov Identifier NCT05321706; estimated study completion in 2027). Determining the optimal timing for initiating treatment is fundamental to optimize the therapy and mitigate potential risks. In addition, it is crucial to explore the use of SGLT2i in HT recipients without diabetes. These data could reveal potential benefits or risks, contributing to more informed therapeutic decisions and improving overall patient management in the post-transplant setting.

## Conclusions

In conclusion, while current findings regarding the safety of SGLT2i are promising, further research is needed to confirm their efficacy in managing LVAD and HT patients. More robust data are required to safely integrate SGLT2i into the clinical management strategies for these vulnerable patient populations.

## Limitations

This review has several limitations, including a limited number of studies with small sample size and heterogeneous methodology. Notably, there was relevant variability in the timing of SGLT2i initiation post-LVAD and HT. Furthermore, the studies had relatively short follow-up periods and provided heterogeneous information on patient characteristics. These limitations affect the generalizability and reliability of the findings, highlighting the need for more extensive and methodologically consistent research, including randomized controlled trials.

## Data Availability

No datasets were generated or analysed during the current study.
